# Sociodemographic profile of tuberculosis and treatment difficulties in light of health literacy

**DOI:** 10.1590/0034-7167-2025-0200

**Published:** 2026-07-24

**Authors:** Dayanne de Nazaré dos Santos, Raquel Gomes da Silva, Erlon Gabriel Rego de Andrade, Ivaneide Leal Ataide Rodrigues, Maria Elizabete de Castro Rassy, Laura Maria Vidal Nogueira

**Affiliations:** I Universidade do Estado do Pará. Belém, Pará, Brazil

**Keywords:** Tuberculosis, Therapeutics, Health Literacy, Primary Health Care, Communicable Diseases., Tuberculosis, Terapéutica, Alfabetización en Salud, Atención Primaria de Salud, Enfermedades Transmisibles.

## Abstract

**Objectives:**

to describe the sociodemographic profile of people with tuberculosis and difficulties in undergoing treatment in Primary Health Care in light of health literacy.

**Methods:**

a cross-sectional study, conducted in four Municipal Health Units located in Belém/Pará/Brazil. A total of 207 people undergoing tuberculosis treatment were interviewed using sociodemographic questionnaire and Health Literacy Test. Data were analyzed using descriptive statistics.

**Results:**

males predominated (n=135/65.2%), in the age groups of 18-29 (n=62/30.0%) and 30-39 years (n=50/24.2%), single (n=97/46.9%), with completed high school/incomplete elementary school (n=70/33.8%; n=63/30.4%) and a family income of two minimum wages (n=108/52.2%). Regarding tuberculosis treatment, they acknowledged difficulties such as removing medication from its packaging, refilling medications, and taking multiple pills simultaneously, highlighting limitations related to health literacy.

**Conclusions:**

investments in structural, human, and material resources should be made by healthcare service professionals/managers and public authorities, aiming to improve the literacy of people with tuberculosis.

## RESUMEN

**Objetivos:**

describir el perfil sociodemográfico de las personas con tuberculosis y las dificultades para llevar a cabo el tratamiento en Atención Primaria de Salud, considerando la alfabetización en salud.

**Métodos:**

estudio transversal en cuatro Unidades de Salud Municipales de Belém/Pará/Brasil. Se entrevistó a 207 personas en tratamiento antituberculoso mediante cuestionario sociodemográfico y Prueba de Alfabetización en Salud. Los datos se analizaron mediante estadística descriptiva.

**Resultados:**

predominó el sexo masculino (n=135/65,2%), en los grupos de edad de 18 a 29 años (n=62/30,0%) y 30 a 39 años (n=50/24,2%), solteros (n=97/46,9%), con educación secundaria completa/primaria incompleta (n=70/33,8%; n=63/30,4%) y un ingreso familiar de dos salarios mínimos (n=108/52,2%). En cuanto al tratamiento de la tuberculosis, reconocieron dificultades como la extracción de medicamentos de su envase, la reposición de medicamentos y la toma simultánea de múltiples pastillas, destacando limitaciones relacionadas con la alfabetización en salud.

**Conclusiones:**

los profesionales y gestores de los servicios sanitarios, así como autoridades públicas, deberían realizar inversiones en recursos estructurales, humanos y materiales para mejorar la alfabetización de personas con tuberculosis.

## INTRODUCTION

Caused by *Mycobacterium tuberculosis*, or Koch’s bacillus, an aerobic and acid-fast microorganism, tuberculosis (TB) remains a significant public health problem, challenging health systems worldwide. Effective control requires achieving a higher cure rate among treated cases and significantly reducing treatment abandonment^(1,2)^. TB remains a significant cause of death globally, affecting 10.8 million people in 2023 and causing 1.25 million deaths, of which 1.09 million occurred among people not infected with the human immunodeficiency virus (HIV). Therefore, treatment becomes a key strategy to reduce both morbidity and mortality associated with TB^(1)^.

Given this scenario, it is worth recognizing that it has been challenging to apply strategic measures to achieve better therapeutic outcomes, especially in countries with the highest disease burdens and low or medium socioeconomic development, such as Brazil^(3)^, whose geopolitical territory includes social disparities, placing it among the 30 countries with the highest burdens of TB and TB/HIV co-infection^(1)^.

In 2024, the country reported 84,308 new cases, resulting in an incidence rate of 39.7 cases/100,000 inhabitants, and in 2023, 6,025 deaths, with a mortality rate of 2.8 deaths/100,000 inhabitants. In the state of Pará, considering the same years, 5,356 new cases and 320 deaths were registered, resulting in incidence and mortality rates of 61.8 cases/100,000 inhabitants and 3.7 deaths/100,000 inhabitants, respectively. In the municipality of Belém, 1,860 new cases and 138 deaths were reported, with incidence and mortality rates of 133.0 cases/100,000 inhabitants and 9.8 deaths/100,000 inhabitants, respectively^(4)^.

Brazil has led collective and intersectoral efforts to control the disease, which is why the Brazilian National Plan to End TB as a Public Health Problem was established^(5)^, based on the End TB Strategy, developed by the World Health Organization (WHO)^(6)^ in the context of the 2030 Agenda for Sustainable Development^(7)^. The plan aims to reduce the incidence of TB by 90% and the deaths from TB by 95% by 2035, resulting in an incidence rate of less than ten cases per 100,000 inhabitants and a mortality rate of less than one death per 100,000 inhabitants^(5)^.

It is known that drug treatment is one of the main control measures, because, when carried out correctly and in a timely manner, it promotes cure and reduces the disease transmission. Aiming for better results in healthcare, directly observed treatment (DOT) was implemented, which is offered by the Brazilian Health System (In Portuguese, *Sistema Único de Saúde* - SUS) throughout the national territory. This consists of observation of a person’s medication intake, and must be carried out by a qualified professional for at least six months^(8)^. This strategy was implemented to contribute to achieving the goals of curing 85% of cases that start treatment and reducing interruption to levels of up to 5%^(1,9)^. However, in Brazil, the percentage of therapeutic interruption among cases confirmed by laboratory criteria, recorded in 2023, was 15.3%^(4)^, above the recommended level.

Treatment adherence is a dynamic and multidimensional process, involving behavioral, psychological, and social aspects. It is necessary to engage individuals and healthcare professionals, especially nurses, to establish bonds that promote effective treatment continuity. Therefore, it is fundamental that professionals provide a welcoming and sensitive listening environment, as well as clear and objective guidance on the actions to be taken by those living with TB, since a lack of understanding of the magnitude of the disease and the importance of proper medication use can lead to treatment discontinuation^(10-12)^.

The implementation of care-educational activities highlights the role of nursing, emphasizing that meeting individual and collective needs is part of professionals’ daily work. Their actions should include family members and other individuals who are in contact with patients, expanding the possibilities for both people with TB and those who share caregiving responsibilities to make informed decisions that contribute to improving their quality of life^(9)^.

In this context, therapeutic self-management by people with TB during the course of treatment is of interest, relating to health literacy (HL), understood here as the knowledge, motivation, and competence of individuals to access, understand, assess, and apply health information to judge and make decisions in daily self-care^(13)^.

Exploring HL is important for empowering and guiding conscious choices, especially in scenarios that imply greater vulnerability for human groups, as occurs in cases of communicable diseases. This is a global concern, as stated in the Shanghai Declaration, which highlights HL as a driver of change at the individual, family, and community levels^(14)^. Therefore, HL is crucial in the pursuit of health actions, as it empowers the population to claim rights and quality services, fostering greater commitment from professionals and public managers in health promotion actions and strategies^(13)^.

Beyond individual and collective empowerment, HL is crucial for achieving satisfactory results in controlling diseases that require prolonged treatment, such as TB. Evidence of this can be seen in a study on HL conducted in one of Pakistan’s districts, which assessed the factors responsible for the low cure rate among people undergoing TB treatment, finding little or no knowledge about the disease^(14)^.

It is considered necessary to propose, implement, and encourage research addressing TB with the use of HL in the Brazilian context, especially in the North region, since HL is essential in healthcare services and for individuals to effectively self-manage their health status, aiming for better TB control^(15,16)^. This reflection is justified by the high magnitude of the disease in the region, marked by high incidence and mortality rates^(4)^.

Research of this nature should primarily take place in Primary Health Care (PHC), a level that plays a fundamental role in the care of people with TB, carrying out work processes inherent to prevention, diagnosis, treatment, and continuous follow-up as part of its responsibilities^(15,16)^.

Given the scientific and social relevance of the topic, it is considered opportune to produce evidence and reflections on treatment, emphasizing, above all, the living conditions and difficulties that people with the disease face in their daily lives. By associating HL with this context, it is possible not only to characterize the reality but also to share results that support new approaches to strengthen adherence to treatment and other control activities, positively impacting the epidemiological scenario.

Recognizing the importance of achieving satisfactory cure and treatment discontinuation rates that meet the WHO and Brazilian Ministry of Health recommendations, the following research question was formulated: what is the sociodemographic profile of people with TB and what difficulties do they face in conducting treatment in PHC services in light of HL?

## OBJECTIVES

To describe the sociodemographic profile of people with tuberculosis and difficulties in undergoing treatment in Primary Health Care in light of health literacy.

## METHODS

### Ethical aspects

Following the guidelines of Resolution 466/2012 of the Brazilian National Health Council/Ministry of Health, which addresses the ethical aspects of conducting research with human subjects in Brazil, approval was obtained from the Research Ethics Committee linked to the Nursing Undergraduate Program at the *Universidade do Estado do Pará*, and institutional authorization was granted by the Municipal Health Department of Belém.

Prior to data collection, the Informed Consent Form (ICF) was read together with participants, who were asked to sign it to declare their formal acceptance. To minimize the risk of compromising their identities, an alphanumeric coding method was adopted, using the letter P for “participant”, followed by an Arabic numeral corresponding to the sequence of interviews.

### Study design and setting

This is a descriptive and cross-sectional study, with a quantitative approach, guided by STrengthening the Reporting of OBservational studies in Epidemiology (STROBE) guidelines^(17)^, made publicly available by Enhancing the QUAlity and Transparency Of health Research (EQUATOR Network).

The study was conducted in the municipality of Belém, capital of the state of Pará, located in northern Brazil, considering its epidemiological importance, as evidenced by the Ministry of Health which, in 2025, identified Belém as the capital city with the highest incidence and mortality rates from TB in the country^(4)^.

For operational purposes, Belém is organized into eight administrative districts: Belém Administrative District (DABEL); Benguí Administrative District (DABEN); Entroncamento Administrative District (DAENT); Guamá Administrative District (DAGUA); Icoaraci Administrative District (DAICO); Mosqueiro Administrative District (DAMOS); Outeiro Administrative District (DAOUT); and Sacramenta Administrative District (DASAC)^(18)^.

The PHC network was chosen as the study setting, more precisely Municipal Health Units (MHUs), selecting four units located in three of the five administrative districts that presented the highest incidences of TB from 2016 to 2020, as demonstrated in the Belém Municipal Health Plan for the four-year period 2022-2025^(19)^. To ensure their anonymity, these units have been identified here as MHU I, MHU II, MHU III, and MHU IV. Among the selected units, MHU I and MHU II are located in DAGUA, MHU III in DAENT, and MHU IV in DASAC.

### Population and selection criteria

In order to define the sample size, the total number of cases that started TB treatment in the aforementioned units from February 2023 onwards and that had regular registration until March 2023, the period in which the statistical survey was carried out, was considered, obtaining a number of 241 cases. Calculation was performed using the Yamane sampling technique^(20)^, with a sampling error of 5%. Thus, 150 was obtained as the minimum number, according to the formula below:


$$n=\frac{N}{1+N\left(e^{2}\right)}$$

Where n = sample size; N = population size; e = constant (0.05).

As for inclusion criteria, the decision was made to select individuals undergoing TB treatment, classified as new cases, following the basic regimen, aged 18 or older, literate, residing in the municipality of Belém, registered at one of the four MHUs, and who had been undergoing treatment for at least two months. Twelve people were excluded because they had cognitive, visual, auditory, and/or oral limitations, as these interfered with their ability to write, read, listen, and/or communicate with the researcher, which made it difficult or impossible to read the instruments used.

Applying these criteria, a total of 207 participants were obtained, exceeding the minimum number considered satisfactory for this study. There were no refusals or withdrawals.

### Study protocol

Data were collected by the principal investigator between June and August 2023 using two instruments. The first was a sociodemographic questionnaire adapted from an instrument with clinical and sociodemographic questions, produced in another study for application among people with TB^(21)^. Originally divided into ten chunks, totaling 121 questions, this instrument addresses various aspects, namely: chunk I (general data); chunk II (personal data); chunk III (household data); chunk IV (socioeconomic data); chunk V (lifestyle habits); chunk VI (contact history); chunk VII (clinical history); chunk VIII (care data); chunk IX (tests performed); and chunk X (perception/knowledge about the disease).

In this study, chunks I, II, and IV were used to investigate general data, personal data, and socioeconomic data related to six variables: one to identify the MHU in which participants were registered; and the other five to understand the sociodemographic profile (sex, age, marital status, education, and monthly family income).

The second instrument is a Health Literacy Test (HLT), adapted for Brazilian Portuguese, aiming to define the level of functional literacy among Brazilian adults. In its full version, the HLT is divided into three parts: the first consists of ten items with quantitative and qualitative responses, and the second consists of three sections (A, B, and C) with multiple-choice answers, according to the situations addressed in each section^(22)^.

In the third part, five items are presented, with quantitative and qualitative responses. Considering the objective of this research and the specificities of the topic, it was decided to use only the first item, as it aims to identify possible difficulties faced by users during treatment, presenting five tasks: “removing the medication from the packaging”; “reading the medication packaging”; “remembering to take the medication”; “replenishing the medication”; and “taking several pills at the same time”. The responses regarding these tasks are identified as “very difficult”, “somewhat difficult”, and “not difficult”^(22)^.

The principal investigator was enrolled in a master’s program in nursing at the proposing institution, receiving training through orientation meetings and regular meetings of a research group at the same institution. Initially, she visited the selected units to understand their realities and introduce herself to the managers and multidisciplinary teams working there, in order to clarify the research objectives and procedures, but also to agree on the availability of a room where activities would be carried out and to request these professionals’ collaboration in whatever else was necessary for the smooth running of the research.

Participants were approached privately while waiting for service at the units, at which time the research was briefly presented to them, inviting them to participate in an individual interview. Prioritizing their comfort and privacy, those who accepted were invited to go to a private room, where only the researcher and the participant were present. The ICF was presented to detail and clarify the research objectives, procedures, risks, and benefits, and they were asked to read and sign it manually to declare their acceptance. The instruments were then applied, beginning with the sociodemographic questionnaire, followed by the third part of HLT.

### Analysis of results and statistics

Data were tabulated in a Microsoft Office Excel^®^ spreadsheet version 2021, and analyzed using the Statistical Package for the Social Sciences version 22.0. The analysis of sociodemographic variables and difficulties related to TB treatment was performed using descriptive statistics, with results expressed as absolute and relative frequencies.

## RESULTS

Among the 207 participants, 69 (33.3%) were registered in MHU I, 54 (26.1%) in MHU II, 56 (27.1%) in MHU III, and 28 (13.5%) in MHU IV. Higher proportions were identified in the male sex, with 135 (65.2%) participants; in the age groups of 18 to 29 and 30 to 39 years, with 62 (30.0%) and 50 (24.2%); among single individuals, with 97 (46.9%); and in the education levels corresponding to completed high school and incomplete elementary school, with 70 (33.8%) and 63 (30.4%), respectively. Finally, the majority of family incomes corresponded to two minimum wages, mentioned by 108 (52.2%) participants, considering the value of R$ 1,320.00 - the wage that was in effect in Brazil in 2023 (Table 1).

**Table 1:** Sociodemographic profile of people undergoing tuberculosis treatment by health unit (N=207), Belém, Pará, Brazil, 2023

Characteristics	MHU I (n=69)	MHU II (n=54)	MHU III (n=56)	MHU IV (n=28)	Total (n=207)
**Sex**					
Male	42 (60.9%)	44 (81.5%)	31 (55.4%)	18 (64.3%)	135 (65.2%)
Female	27 (39.1%)	10 (18.5%)	25 (44.6%)	10 (35.7%)	72 (34.8%)
**Age group**					
18 to 29 years	26 (37.7%)	18 (33.3%)	12 (21.4%)	6 (21.4%)	62 (30.0%)
30 to 39 years	12 (17.4%)	12 (22.2%)	20 (35.7%)	6 (21.4%)	50 (24.2%)
40 to 49 years	12 (17.4%)	3 (5.6%)	6 (10.7%)	4 (14.3%)	25 (12.1%)
50 to 59 years	8 (11.6%)	13 (24.1%)	16 (28.6%)	9 (32.1%)	46 (22.2%)
60 to 84 years	11 (15.9%)	8 (14.8%)	2 (3.6%)	3 (10.7%)	24 (11.6%)
**Marital status**					
Single	32 (46.4%)	22 (40.7%)	29 (51.8%)	14 (50.0%)	97 (46.9%)
Married	32 (46.4%)	21 (38.9%)	24 (42.9%)	12 (42.9%)	89 (43.0%)
Separated	3 (4.3%)	3 (5.6%)	1 (1.8%)	1 (3.6%)	8 (3.9%)
Widow	2 (2.9%)	8 (14.8%)	2 (3.6%)	1 (3.6%)	13 (6.3%)
**Education**					
Incomplete elementary school	25 (36.2%)	15 (27.8%)	15 (26.8%)	8 (28.6%)	63 (30.4%)
Complete elementary school	10 (14.5%)	11 (20.4%)	11 (19.6%)	4 (14.3%)	36 (17.4%)
Incomplete high school	6 (8.7%)	4 (7.4%)	6 (10.7%)	4 (14.3%)	20 (9.7%)
Complete high school	24 (34.8%)	17 (31.5%)	20 (35.7%)	9 (32.1%)	70 (33.8%)
Incomplete higher education	3 (4.3%)	1 (1.9%)	2 (3.6%)	1 (3.6%)	7 (3.4%)
Complete higher education	1 (1.4%)	6 (11.1%)	2 (3.6%)	2 (7.1%)	11 (5.3%)
**Monthly family income**					
Unemployed	0 (0.0%)	1 (1.9%)	0 (0.0%)	0 (0.0%)	1 (0.5%)
One wage	2 (2.9%)	5 (9.3%)	0 (0.0%)	0 (0.0%)	7 (3.4%)
Two wages	39 (56.5%)	16 (29.6%)	38 (67.9%)	15 (53.6%)	108 (52.2%)
Three wages	25 (36.2%)	15 (27.8%)	15 (26.8%)	9 (32.1%)	64 (30.9%)
Four wages	3 (4.3%)	11 (20.4%)	3 (5.4%)	4 (14.3%)	21 (10.1%)
Five wages	0 (0.0%)	4 (7.4%)	0 (0.0%)	0 (0.0%)	4 (1.9%)
Six wages	0 (0.0%)	2 (3.7%)	0 (0.0%)	0 (0.0%)	2 (1.0%)

*MHU - Municipal Health Unit.*

Concerning difficulties related to treatment, although 98 (47.3%) participants stated that “removing the medication from the packaging” is not a difficult task, 90 (43.5%) considered it somewhat difficult. The tasks “reading the medication packaging”, “remembering to take the medication”, and “replenishing the medication” were considered somewhat difficult by 147 (71.0%), 167 (80.7%), and 105 (50.7%) participants, respectively. While 109 (52.7%) stated that “taking several pills at the same time” is not a difficult task, 34 (16.4%) considered it very difficult, representing the highest percentage for this response among the difficulties mentioned (Table 2).

**Table 2:** Difficulties related to treatment, reported by people undergoing treatment for tuberculosis (N=207), Belém, Pará, Brazil, 2023

Difficulties mentioned	Very difficult	Somewhat difficult	Not difficult
Removing the medication from the packaging	19 (9.2%)	90 (43.5%)	98 (47.3%)
Reading the medication packaging	5 (2.4%)	147 (71.0%)	55 (26.6%)
Remembering to take the medication	1 (0.5%)	167 (80.7%)	39 (18.8%)
Replenishing the medications	18 (8.7%)	105 (50.7%)	84 (40.6%)
Taking several pills at the same time	34 (16.4%)	64 (30.9%)	109 (52.7%)

## DISCUSSION

Participants were predominantly men aged 18 to 39, single, with low levels of education and a monthly family income equivalent to two minimum wages. They acknowledged difficulties in carrying out TB treatment, such as removing the medication from its packaging, refilling the medication, and taking multiple pills at the same time.

The predominant sex is consistent with the literature, as it is expressed in official data from the WHO and the Ministry of Health^(1,4)^, as well as in several national^(3,23)^ and international^(24,25)^ studies, confirming higher proportions of TB cases in this population. In 2024, of the 84,308 new cases registered in Brazil, 57,506 (68.2%) occurred in males, whose incidence rates, when compared to those of females, were higher in all age groups, except in the 10 to 14 year age group^(4)^.

Focusing on the local reality, it is noteworthy that, from 2009 to 2016, when assessing the cases reported in Belém, an ecological study pointed to 11,103 new cases, with an incidence rate of 97.5 cases/100,000 inhabitants, an average age of 38.6 years and a standard deviation of 17.1, showing that the incidence rate was higher in the male population (12.4 cases/10,000 men) than in the female population (7.3 cases/10,000 women)^(26)^.

It is possible to infer that this epidemiological overview is strongly related to the culture concerning men’s health, as studies have identified reduced or limited demand from this population for services offered in PHC^(27-29)^, translating into an aggravating factor for high rates of morbidity and mortality from various causes. In many contexts, men’s demand for these services is substantially lower than that of women, in addition to showing low adherence to therapeutic proposals and proposals for health promotion and disease prevention^(30)^.

Thus, HL’s fragility is noticeable in a large part of the male population, since people with better HL levels tend to recognize, more easily, the importance of preventive and health-maintenance actions, in addition to adopting self-care practices^(31)^. It is understood that the absence of this public in healthcare services makes them more vulnerable to illness, in addition to delaying diagnoses that could be made early and the corresponding therapeutic interventions, reflecting low competence and skill of HL.

In the scenario of greater illness in the male population, men’s limitations in attending healthcare services can be explained by several reasons, such as historical and sociocultural factors that have contributed and still contribute to producing outdated thought patterns around masculinity, linking men to the notion of invulnerability^(29,32,33)^.

Comparing the inequalities between men and women in the context of TB, such as social status, economic position, and access to various resources, the differences attributed to economic and sociocultural factors are notable, impacting adherence to TB treatment^(34)^.

Concerning age range, the results indicate that the predominant group was between 18 and 39 years old, approaching the data published in the TB Epidemiological Bulletin for the year 2025, which shows the incidence data for 2024. This document shows that, in both sexes, the highest incidence rates were identified in the 20 to 34 age group, with an incidence ratio between sexes equal to 2.18, demonstrating that, in addition to men, those most affected are in the economically active age group^(4)^, as evidenced in the literature^(35,36)^.

It is worth highlighting that younger people have greater potential for HL, enabling them to identify and access reliable sources of information. This gives them a better chance of obtaining quality information and implementing it to promote self-care^(37)^. This consideration is made taking into account that the older the person, the greater the potential for vulnerabilities surrounding HL, as aging is more likely to affect cognition and special senses such as hearing and vision, interfering with the understanding of health information and other topics. Furthermore, older adults commonly rely on third-party assistance for daily care, with these third parties receiving and implementing information without an older adult actively participating in decision-making processes, often assigning them a secondary role in care^(31)^.

Data regarding marital status show that there was no significant difference in the number of single and married people, or in the number of separated and widowed people. Despite this, it is possible to infer that this variable may reflect the spatial mobility of TB, resulting in higher or lower levels of social contact with romantic/sexual partners, since proximity of contact favors transmission. An international study indicated that marital status (single, widowed, or divorced) reflects the degree of family support during treatment, strengthening or reducing episodes of psychosocial stress through interpersonal relationships^(38)^.

Research indicated an association between TB and education level in Belém, a scenario in which a portion of participants had up to four years of schooling^(26)^. It is known that less educated people express greater vulnerability, as they have greater difficulty accessing and understanding health information, resulting in implications for HL and, consequently, less ability to identify risks of TB transmission, in addition to influencing adherence to treatment^(2,39)^.

However, assessing HL based solely on educational level does not guarantee the accuracy of the findings, since years of formal education are quantified without considering the knowledge acquired through the subject’s varied experiences. For instance, an individual may possess numerical and reading comprehension skills, but have difficulty applying them in a healthcare context, demonstrating limitations related to HL. Therefore, a high level of education does not necessarily result in satisfactory HL^(40)^.

Another study, conducted in the same municipality, indicated that 91% of participants had a family income of less than one minimum wage^(41)^. In turn, in the state of Bahia, it was indicated that the majority of people with TB (80.26%) had a monthly income of less than R$ 1,000.00, considering their main source of income, emphasizing that the disease and poverty conditions are interconnected, a fact that results in worse health conditions for these people^(42)^. In terms of formal education and wages, the literature shows that educational level impacts employment opportunities and, consequently, individual and family income. Thus, the more favorable the socioeconomic conditions, the greater the chances of having satisfactory HL and good health^(43)^.

It is worth highlighting that the 2030 Agenda foresees, among the 17 Sustainable Development Goals developed by the United Nations, the eradication of poverty in all its forms and in all places, in order to create the necessary conditions to promote a dignified life for all^(7)^. When relating these aspects to the current multi-determined scenario of the capital of Pará and to the number of TB cases in this territory^(4)^, the need to propose and implement interventional measures beyond the biological field, also encompassing the social and educational fields, becomes evident.

Regarding the difficulties in carrying out treatment, it is understood that the difficulty in removing the medication from its packaging can be overcome through the initiative of the professional responsible for DOT in assisting users with this task which, although simple, is fundamental to consolidating the treatment, by delivering pills along with a glass of water to ensure ingestion. Thus, a professional provides personalized assistance, valuing the limitations of each individual to meet their needs^(2)^.

The difficulty in reading medication packaging may be related to factors such as lower cognitive ability, reading restrictions, and the complexity of the information contained on the packaging^(44)^. Furthermore, it is known that, conversely, a lack of understanding of written information can interfere with users’ autonomy and empowerment, as well as contribute to the incorrect application of information shared by healthcare professionals, resulting in unsatisfactory outcomes and ineffective use of healthcare services^(39)^.

Finally, difficulties in remembering to take and replenish medications may be related to socioeconomic factors, considering the established therapeutic regimen for TB, which consists of the observed administration of medications according to MHUs’ routine, which may be carried out every weekday or at least three times a week^(2,45)^. Thus, difficulties arise, especially for users who are employed, who often choose not to attend health units for treatment due to fear of wage loss, unemployment, and the consequent reduction in family income^(46)^.

The therapeutic regimen for TB is complex, which is why it can constitute a barrier to adherence to treatment when there is a lack or deficiency of understanding about its importance. In this regard, it is evident that HL is fundamental to tackling TB. A study that assessed the HL of contacts of people undergoing treatment for pulmonary TB identified a low level of HL, concluding that the lack or deficiency of basic information about the disease constituted a risk of infection by *Mycobacterium tuberculosis* and possible illness^(13)^. Another study demonstrated that people with low HL levels are more susceptible to medication non-adherence, as these levels are related to a lack of understanding of health information^(47)^.

Analyzing the difficulties reported by participants in this study, it became clear that there is a need to implement strategies that minimize the potential repercussions caused by these difficulties. In this sense, nurses play a crucial role in helping users overcome these difficulties, as they are recognized for their ability to holistically understand the human being, act with a focus on comprehensive care, and address vulnerabilities to transform them into strengths^(9)^.

Several studies have reported the participation or contributions of nursing in strengthening TB control actions, among which the care of people with the disease stands out^(48-51)^. A comprehensive scoping review, which included 40 national and international studies, identified nursing actions that promote adherence to TB treatment, organizing them into two thematic categories: one on care directed at the specific needs of these individuals; and another on how nursing professionals address the social determinants of health^(52)^.

Characterized by preparing and monitoring their multidisciplinary teams to promote HL, Health Literate Organizations encourage the implementation of strategies that, by involving users, professionals, and institutions, direct care towards individuals, increasing its effectiveness. It is known that addressing individualized needs is essential for conducting treatment, as it establishes bonds and trust, generating positive health outcomes^(9,39)^. Thus, to promote HL, it is necessary for nurses to adopt appropriate strategies, such as clear and objective communication, in addition to the possibility of using easy-to-apply technologies^(39)^.

This demonstrates that knowing the difficulties related to TB treatment is essential, as it makes it possible to reflect critically and propose coordinated actions between managers, healthcare professionals and users, directed to meet individual and collective needs, contributing to avoiding therapeutic interruption and strengthening disease control^(47)^.

### Study limitations

Limitations are related to the fact that the study was conducted in health units of the PHC located in only three districts in the municipality of Belém, reflecting the specific realities of healthcare. Epidemiological, geographic, socioeconomic, and territorial characteristics may have substantially influenced the data, as these factors are related to the production of knowledge and practices, thus impacting HL to strengthen or reduce the difficulties surrounding TB treatment.

To expand evidence on the subject and better understand it, it is essential that new studies be carried out, aiming to encompass a greater number of units and other districts in this municipality, as well as a greater number of municipalities in the North region and other regions of the national territory. This consideration aims to incorporate other levels of health care, given that people with TB are attended to and treated in PHC, in secondary/specialized care, and in tertiary/hospital care according to their clinical characteristics and the therapeutic support needs arising from these characteristics.

### Contributions to nursing, health, or public policy

Given its relevance, this study contributes to the fields of nursing and public health by revealing the profile of people with TB and the difficulties surrounding the disease treatment. Thus, it highlighted the need to update, restructure, or reinforce users’ knowledge and practices through educational actions and strategies in daily healthcare settings, aiming to strengthen HL as a fundamental element in facing and overcoming these difficulties.

This demonstrates that content inherent to HL should permeate students’ training processes in higher education, such as nursing students, and professionals’ training processes in continuing education and permanent education, such as nursing teams, especially nurses. They must lead these teams with ethical, scientific, technical, and social responsibility, relating strongly with the other members of the interdisciplinary and multiprofessional teams, as guided by the principles of universality, comprehensiveness, and equity, which are the doctrinal principles of SUS.

The results and subsequent discussions can stimulate reflection among students, professionals and managers, public authorities, users of healthcare services, and other segments of organized civil society. This mobilization constitutes opportunities for public health and education policies to be proposed or rethought, especially at the local or regional levels, to improve assistance to human groups, the management of these services, teaching, research, and political participation in governance and decision-making spheres that affect these groups.

## CONCLUSIONS

It was identified that the sociodemographic profile was primarily composed of males, aged 18 to 39, single, with low levels of education and low monthly family income. In TB treatment, difficulties were evident, such as removing medication from its packaging, replenishing medication, and taking multiple pills simultaneously, indicating that investments in various resources, such as structural, human, and material resources, should be made by healthcare service professionals and managers, as well as by public authorities, according to their governance capabilities, aiming to improve PHC.

These investments need to strengthen users’ HL so that the knowledge and practices they develop daily enhance the chances of achieving a cure and controlling the disease, both in the municipality of Belém and, more broadly, in the Amazon region and throughout the national territory. This consideration is made considering that, despite the evolution of technical and scientific knowledge and being the target of health policies and programs over the years, TB still plagues individuals and groups, limiting their quality of life and the possibilities for improving it.

For this reason, a long road still needs to be traveled to overcome TB as a significant public health problem, a context in which HL expresses robust possibilities for these people to reflect on their living and health conditions and the need to transform, to some degree, the ways they think and act, how they obtain health information, how they value and assess its relevance, how they apply and share it, and the explanations that underlie and justify such ways and the choices in their environment.

Investments that enable us to understand the sociodemographic profile of people with TB in different social and territorial realities are fundamental to characterizing the individual and collective needs that arise from this profile. This makes it possible to better target the control of the disease in the municipalities and states of the federation, especially in those whose geographical characteristics imply great challenges, as is the particular case of the municipalities and states of the Amazon region.

Part of control actions and strategies should focus on strengthening users’ HL, through qualified professional guidance and democratic access to technological products that guide health education processes in PHC. This aims to empower people with TB to develop their self-care in a rational, sustainable, and dialogical way, i.e., based on critical-reflective attitudes resulting from the fruitful interaction between them and professionals.

This interaction should not only contribute to the sharing of knowledge, practices, and experiences, but also strengthen both the dialogues between scientific knowledge, common sense, and the bonds, which generate trust and communication, as these are aspects that intertwine with HL and influence health-related phenomena. Therefore, with actions and strategies that value HL and the collective dimension of illness, it is understood that individuals can better face TB as a socially determined disease, highlighting the importance of studies such as this one.

## Data Availability

The research data are available only upon request.
