# Supplementary material for: Sociodemographic profile of tuberculosis and treatment difficulties in light of health literacy
Source: Rev Bras Enferm. 2026 Jul 24;79:e20250200. doi: 10.1590/0034-7167-2025-0200 (PMC13411002; doi:10.1590/0034-7167-2025-0200)
Supplement: Translation

# Translation: Perfil sociodemográfico da tuberculose e dificuldades no tratamento à luz do letramento em saúde

**DOI:** 10.1590/0034-7167-2025-0200pt

**Authors:** Dayanne de Nazaré dos Santos, Raquel Gomes da Silva, Erlon Gabriel Rego de Andrade, Ivaneide Leal Ataide Rodrigues, Maria Elizabete de Castro Rassy, Laura Maria Vidal Nogueira

**Affiliations:** I Universidade do Estado do Pará. Belém, Pará, Brasil

**Keywords:** Tuberculose, Terapêutica, Letramento em Saúde, Atenção Primária à, Saúde, Doenças Transmissíveis.

## Abstract

**Objetivos:**

descrever o perfil sociodemográfico de pessoas com tuberculose e dificuldades para realizar o tratamento na Atenção Primária à Saúde à luz do letramento em saúde.

**Métodos:**

estudo transversal, realizado em quatro Unidades Municipais de Saúde, localizadas em Belém/Pará/Brasil. Foram entrevistadas 207 pessoas em tratamento para tuberculose, utilizando questionário sociodemográfico e Teste de Letramento em Saúde. Dados foram analisados por estatística descritiva.

**Resultados:**

predominaram homens (n=135/65,2%), nas faixas etárias de 18-29 (n=62/30,0%) e 30-39 anos (n=50/24,2%), solteiros (n=97/46,9%), com ensino médio completo/ensino fundamental incompleto (n=70/33,8%; n=63/30,4%) e renda familiar de dois salários mínimos (n=108/52,2%). No tratamento para tuberculose, reconheceram dificuldades como retirar o medicamento da embalagem, repor medicamentos e tomar vários comprimidos ao mesmo tempo, configurando limitações relacionadas ao letramento em saúde.

**Conclusões:**

investimentos em recursos estruturais, humanos e materiais devem ser realizados por profissionais/gestores dos serviços de saúde e autoridades públicas, visando qualificar o letramento de pessoas com tuberculose.

## INTRODUÇÃO

Causada pelo *Mycobacterium tuberculosis* ou bacilo de Koch, microrganismo aeróbio e álcool-ácido resistente, a tuberculose (TB) ainda configura relevante problema de saúde pública, pois desafia sistemas de saúde em todo o mundo. Seu controle efetivo perpassa a necessidade de obter maior proporção de cura entre os casos em tratamento e maior redução do abandono terapêutico^(1,2)^. Permanece como importante causa de óbito no cenário global, visto que, em 2023, acometeu 10,8 milhões de pessoas e gerou 1,25 milhão de óbitos, dos quais 1,09 milhão ocorreram entre pessoas não infectadas pelo vírus da imunodeficiência humana (HIV). Por isso, a cura torna-se uma estratégia para reduzir tanto a morbidade quanto a mortalidade associadas à TB^(1)^.

Diante desse panorama, é oportuno reconhecer que tem sido desafiador aplicar medidas estratégicas para alcançar melhores resultados terapêuticos, sobretudo nos países que detêm as maiores cargas de adoecimento e apresentam baixo ou médio desenvolvimento socioeconômico, a exemplo do Brasil^(3)^, cujo território geopolítico contempla disparidades sociais, posicionando-o entre os 30 países com as maiores cargas de TB e da coinfecção TB/HIV^(1)^.

Em 2024, o país notificou 84.308 casos novos, resultado no coeficiente de incidência de 39,7 casos/100 mil habitantes e, em 2023, 6.025 óbitos, com coeficiente de mortalidade de 2,8 óbitos/100 habitantes. No estado do Pará, considerando os mesmos anos, foram registrados 5.356 casos novos e 320 óbitos, resultando nos coeficientes de incidência e de mortalidade iguais a 61,8 casos/100 mil habitantes e 3,7 óbitos/100 mil habitantes. Por sua vez, no município de Belém, foram notificados 1.860 casos novos e 138 óbitos, com coeficientes de incidência e de mortalidade iguais a 133,0 casos/100 mil habitantes e 9,8 óbitos/100 mil habitantes^(4)^.

O Brasil tem liderado esforços coletivos e intersetoriais para controlar a doença, motivo pelo qual foi instituído o Plano Nacional pelo Fim da TB como Problema de Saúde Pública^(5)^, baseado na Estratégia pelo Fim da TB (*End TB Strategy*), elaborada pela Organização Mundial da Saúde (OMS)^(6)^ no contexto da Agenda 2030 para o desenvolvimento sustentável^(7)^. O plano tem como metas, até 2035, reduzir 90% da incidência e 95% dos óbitos por TB, repercutindo no coeficiente de incidência menor que dez casos/100 mil habitantes e no coeficiente de mortalidade menor que um óbito/100 mil habitantes^(5)^.

Sabe-se que o tratamento medicamentoso é uma das principais medidas de controle, pois, quando realizado corretamente e em tempo oportuno, promove a cura e reduz a transmissão da doença. Visando melhores resultados na atenção à saúde, implantou-se o tratamento diretamente observado (TDO), o qual é ofertado pelo Sistema Único de Saúde (SUS) em todo o território nacional, consistindo na observação relativa à ingestão dos medicamentos pela pessoa em tratamento, devendo ser realizada por profissional habilitado durante, no mínimo, seis meses^(8)^. Essa estratégia foi implantada para contribuir com o alcance das metas de curar 85% dos casos que iniciem tratamento e reduzir a interrupção a patamares de até 5%^(1,9)^. Todavia, no Brasil, o percentual de interrupção terapêutica entre os casos confirmados por critério laboratorial, registrado em 2023, foi de 15,3%^(4)^, acima do recomendado.

A adesão ao tratamento é um processo dinâmico e multidimensional, pois envolve aspectos comportamentais, psíquicos e sociais, sendo necessário engajar o indivíduo e os profissionais de saúde, especialmente de enfermagem, para estabelecer vínculos que propiciem a continuidade do tratamento de modo eficaz. Portanto, é fundamental que os profissionais acolham e promovam escuta sensível, além de orientar, clara e objetivamente, as condutas a serem adotadas pelos que convivem com a TB, pois a falta ou carência de entendimento sobre a magnitude da doença e a importância do uso adequado dos medicamentos pode resultar na sua descontinuidade^(10-12)^.

A realização de atividades cuidativo-educacionais destaca a enfermagem, ao apontar o atendimento às necessidades individuais e coletivas como parte do fazer diário dos seus profissionais. Suas ações devem incluir familiares e outras pessoas que sejam comunicantes, ampliando as possibilidades para que tanto as pessoas com TB quanto as que com elas partilham ações de cuidado tomem decisões assertivas e que contribuam para fortalecer a qualidade de vida^(9)^.

Nesse contexto, desperta interesse a autogestão terapêutica por pessoas com TB no curso do tratamento, remetendo ao letramento em saúde (LS), aqui entendido como conhecimento, motivação e competência das pessoas em ter acesso, compreender, avaliar e aplicar informações sobre saúde para julgar e tomar decisões no cotidiano do autocuidado^(13)^.

Explorar o LS é importante para instrumentalizar e guiar escolhas conscientes, sobretudo em cenários que implicam maior vulnerabilidade aos grupos humanos, como ocorre em casos de doenças transmissíveis. Trata-se de uma preocupação mundial, como explicitado na Declaração de Xangai, que ressalta o LS como propulsor de mudanças em nível individual, familiar e comunitário^(14)^. Portanto, o LS é determinante na busca por ações de saúde, uma vez que empodera a população para reivindicar direitos e serviços de qualidade, favorecendo maior comprometimento dos profissionais e dos gestores públicos nas ações e estratégias de promoção da saúde^(13)^.

Além do empoderamento individual e coletivo, o LS é determinante para alcançar resultados satisfatórios no controle de doenças que exigem tratamento prolongado, como a TB. Evidência disso pode ser verificada em estudo acerca do LS, realizado em um dos distritos do Paquistão, o qual avaliou os fatores responsáveis pela baixa proporção de cura entre pessoas em tratamento para TB, constatando-se pouco ou nenhum conhecimento sobre a doença^(14)^.

Considera-se que é necessário propor, implementar e estimular pesquisas que abordem a TB com o LS no contexto brasileiro, especialmente na região Norte, posto que o LS é essencial nos serviços de saúde e para que os indivíduos autogerenciem efetivamente seu estado de saúde, visando ao melhor controle da TB^(15,16)^. Essa reflexão se justifica pela alta magnitude da doença na região, marcada por elevados coeficientes de incidência e de mortalidade^(4)^.

Pesquisas com essa natureza devem ocorrer sobretudo na Atenção Primária à Saúde (APS), nível que desempenha papel fundamental nos cuidados às pessoas com TB, realizando processos de trabalho inerentes à prevenção, ao diagnóstico, ao tratamento e ao acompanhamento contínuo como parte de suas atribuições^(15,16)^.

Diante da relevância científica e social do tema, considera-se oportuno produzir evidências e reflexões acerca do tratamento, enfatizando, sobretudo, as condições de vida e as dificuldades que pessoas com a doença enfrentam no cotidiano. Ao associar o LS para descrever esse contexto, pode-se não apenas caracterizar a realidade, mas também compartilhar resultados que subsidiem novas abordagens para fortalecer a adesão ao tratamento e outras atividades de controle, repercutindo positivamente no cenário epidemiológico.

Reconhecendo a importância de alcançar proporções de cura e interrupção terapêutica satisfatórias e que atendam às recomendações da OMS e do Ministério da Saúde do Brasil, formulou-se a questão de pesquisa: qual o perfil sociodemográfico de pessoas com TB e quais dificuldades enfrentam para conduzir o tratamento em serviços da APS à luz do LS?

## OBJETIVOS

Descrever o perfil sociodemográfico de pessoas com tuberculose e dificuldades para realizar o tratamento na Atenção Primária à Saúde à luz do letramento em saúde.

## MÉTODOS

### Aspectos éticos

Seguindo as orientações da Resolução nº 466/2012 do Conselho Nacional de Saúde/Ministério da Saúde, que trata dos aspectos éticos para desenvolver pesquisas com seres humanos no Brasil, obtiveram-se a aprovação do Comitê de Ética em Pesquisa vinculado ao Curso de Graduação em Enfermagem da Universidade do Estado do Pará e a autorização institucional da Secretaria Municipal de Saúde de Belém.

Precedendo à coleta dos dados, realizou-se a leitura do Termo de Consentimento Livre e Esclarecido (TCLE) com os participantes, solicitando que o assinassem para declarar o aceite formal. Visando minimizar o risco de quebra de sigilo das suas identidades, adotou-se o método de codificação alfanumérica, utilizando a letra P, de “participante”, seguida por número arábico correspondente à sequência das entrevistas.

### Desenho e cenário do estudo

Estudo descritivo e transversal, com abordagem quantitativa, norteado pelas orientações do instrumento *STrengthening the Reporting of OBservational studies in Epidemiology* (STROBE)^(17)^, disponibilizado publicamente pela *Enhancing the QUAlity and Transparency Of health Research* (EQUATOR *Network*).

Foi realizado no município de Belém, capital do estado do Pará, localizado na região Norte do Brasil, considerando sua importância epidemiológica, evidenciada pelo Ministério da Saúde que, em 2025, apontou Belém como a capital que apresentou os maiores coeficientes de incidência e de mortalidade por TB no território nacional^(4)^.

Para fins operacionais, Belém se organiza em oito distritos administrativos, sendo eles: Distrito Administrativo de Belém (DABEL); Distrito Administrativo do Benguí (DABEN); Distrito Administrativo do Entroncamento (DAENT); Distrito Administrativo do Guamá (DAGUA); Distrito Administrativo de Icoaraci (DAICO); Distrito Administrativo de Mosqueiro (DAMOS); Distrito Administrativo de Outeiro (DAOUT); e Distrito Administrativo da Sacramenta (DASAC)^(18)^.

Como cenário, elegeu-se a rede da APS, mais precisamente as Unidades Municipais de Saúde (UMSs), optando-se por quatro unidades localizadas em três dos cinco distritos administrativos que apresentaram as maiores incidências de TB no período de 2016 a 2020, como demonstrado no Plano Municipal de Saúde de Belém para o quadriênio 2022-2025^(19)^. Garantindo seu anonimato, essas unidades foram aqui identificadas como UMS I, UMS II, UMS III e UMS IV. Entre as unidades selecionadas, a UMS I e a UMS II estão localizadas no DAGUA; a UMS III, no DAENT; e a UMS IV, no DASAC.

### População e critérios de seleção

Visando definir o número amostral, considerou-se o total de casos que iniciaram tratamento para TB nas referidas unidades a partir de fevereiro de 2023 e que estavam com registro regular até março de 2023, período em que o levantamento estatístico foi executado, obtendo-se o número de 241 casos. O cálculo foi realizado por meio da técnica de amostragem de Yamane^(20)^, com erro amostral de 5%. Assim, obteve-se 150 como número mínimo, segundo a fórmula abaixo:


$$n=\frac{N}{1+N\left(e^{2}\right)}$$

Onde n = tamanho da amostra; N = tamanho da população; e = constante (0,05).

Quanto aos critérios de inclusão, optou-se por pessoas que estavam em tratamento para TB, enquadradas como casos novos, realizando o esquema básico, com idade igual ou superior a 18 anos, que sabiam ler e escrever, residentes no município de Belém, cadastradas em uma das quatro UMSs e que estivessem com, no mínimo, dois meses de tratamento. Foram excluídas 12 pessoas que apresentaram limitações cognitiva, visual, auditiva e/ou de oralidade, pois interferiam na capacidade de escrever, ler, ouvir e/ou comunicar-se com a pesquisadora, motivo pelo qual dificultavam ou impediam a leitura dos instrumentos utilizados.

Aplicando esses critérios, obteve-se o total de 207 participantes, excedendo o número mínimo, considerado satisfatório para este estudo. Não houve recusas ou desistências.

### Protocolo do estudo

Os dados foram coletados pela pesquisadora principal, no período de junho a agosto de 2023, utilizando dois instrumentos. O primeiro corresponde a um questionário sociodemográfico adaptado de um instrumento com perguntas de natureza clínica e sociodemográfica, produzido em outro estudo para ser aplicado entre pessoas com TB^(21)^. Originalmente dividido em dez blocos, totalizando 121 perguntas, esse instrumento aborda diversos aspectos, sendo eles: bloco I (dados gerais); bloco II (dados pessoais); bloco III (dados de domicílio); bloco IV (dados socioeconômicos); bloco V (hábitos de vida); bloco VI (história de contato); bloco VII (história clínica); bloco VIII (dados sobre atendimento); bloco IX (exames realizados); e bloco X (percepção/conhecimento sobre a doença).

Neste estudo, foram empregados os blocos I, II e IV para investigar dados gerais, dados pessoais e dados socioeconômicos referentes a seis variáveis: uma para identificar a UMS na qual o participante estava cadastrado; e as outras cinco para conhecer o perfil sociodemográfico (sexo, idade, estado civil, escolaridade e renda familiar mensal).

O segundo instrumento corresponde ao Teste de Letramento em Saúde (TLS), adaptado para a língua portuguesa do Brasil, visando definir o nível de letramento funcional entre adultos brasileiros. Em sua versão integral, o TLS é dividido em três partes, sendo a primeira constituída por dez itens, com respostas quantitativas e qualitativas, e a segunda por três trechos (A, B e C), com respostas de múltipla escolha, de acordo com as situações tratadas em cada trecho^(22)^.

Na terceira parte, são apresentados cinco itens, com respostas quantitativas e qualitativas. Considerando o objetivo desta pesquisa e as especificidades do tema, optou-se por utilizar somente o primeiro item, pois visa identificar possíveis dificuldades enfrentadas pelos usuários durante o tratamento, apresentando cinco tarefas: “retirar o medicamento da embalagem”; “ler a embalagem do medicamento”; “lembrar de tomar o medicamento”; “repor os medicamentos”; e “tomar vários comprimidos ao mesmo tempo”. As respostas acerca dessas tarefas são identificadas como “muito difícil”, “pouco difícil” e “não é difícil”^(22)^.

A pesquisadora principal estava matriculada no curso de mestrado em enfermagem da instituição proponente, sendo capacitada em reuniões de orientação e nos encontros regulares de um grupo de pesquisa da mesma instituição. Inicialmente, visitou as unidades selecionadas para conhecer suas realidades e se apresentar aos gestores e às equipes multiprofissionais que nelas atuavam, no intuito de esclarecê-los acerca dos objetivos e procedimentos da pesquisa, mas também para pactuar a liberação de uma sala na qual as atividades seriam realizadas e solicitar a colaboração desses profissionais no que mais fosse necessário para o bom andamento da pesquisa.

Os participantes foram abordados de maneira particular enquanto aguardavam atendimento nas dependências das unidades, ocasião em que a pesquisa lhes foi apresentada brevemente, convidando-os a participar de uma entrevista individual. Prezando por seu conforto e privacidade, os que aceitaram foram convidados a se dirigir à sala reservada, ambiente no qual estavam somente a pesquisadora e o participante. O TCLE foi apresentado para detalhar e esclarecer os objetivos, procedimentos, riscos e benefícios da pesquisa, solicitando-se ao participante que o lesse e o assinasse manualmente para declarar o aceite. Os instrumentos foram aplicados em seguida, iniciando pelo questionário sociodemográfico, passando-se à terceira parte do TLS.

### Análise dos resultados e estatística

Os dados foram tabulados em planilha do *Microsoft Office Excel^®^*, versão 2021, e analisados com auxílio do *software Statistical Package for the Social Science*, versão 22.0. A análise das variáveis sociodemográficas e das dificuldades relacionadas ao tratamento para TB ocorreu por meio da estatística descritiva, cujos resultados estão expressos em frequências absolutas e relativas.

## RESULTADOS

Entre os 207 participantes, 69 (33,3%) estavam cadastrados na UMS I; 54 (26,1%), na UMS II; 56 (27,1%), na UMS III; e 28 (13,5%), na UMS IV. Maiores proporções foram identificadas no sexo masculino, com 135 (65,2%) participantes; nas faixas etárias de 18 a 29 e 30 a 39 anos, com 62 (30,0%) e 50 (24,2%); entre solteiros, com 97 (46,9%); e na escolaridade referente ao ensino médio completo e ao ensino fundamental incompleto, com 70 (33,8%) e 63 (30,4%), respectivamente. Por fim, a renda familiar majoritária correspondeu a dois salários mínimos, mencionados por 108 (52,2%) participantes, considerando o valor de R$ 1.320,00 - salário que vigorava, em 2023, no território brasileiro (Tabela 1).

**Tabela 1:** Perfil sociodemográfico de pessoas em tratamento para tuberculose por unidade de saúde (N=207), Belém, Pará, Brasil, 2023

Características	UMS I (n=69)	UMS II (n=54)	UMS III (n=56)	UMS IV (n=28)	Total (n=207)
**Sexo**					
Masculino	42 (60,9%)	44 (81,5%)	31 (55,4%)	18 (64,3%)	135 (65,2%)
Feminino	27 (39,1%)	10 (18,5%)	25 (44,6%)	10 (35,7%)	72 (34,8%)
**Faixa etária**					
18 a 29 anos	26 (37,7%)	18 (33,3%)	12 (21,4%)	6 (21,4%)	62 (30,0%)
30 a 39 anos	12 (17,4%)	12 (22,2%)	20 (35,7%)	6 (21,4%)	50 (24,2%)
40 a 49 anos	12 (17,4%)	3 (5,6%)	6 (10,7%)	4 (14,3%)	25 (12,1%)
50 a 59 anos	8 (11,6%)	13 (24,1%)	16 (28,6%)	9 (32,1%)	46 (22,2%)
60 a 84 anos	11 (15,9%)	8 (14,8%)	2 (3,6%)	3 (10,7%)	24 (11,6%)
**Estado civil**					
Solteiro	32 (46,4%)	22 (40,7%)	29 (51,8%)	14 (50,0%)	97 (46,9%)
Casado	32 (46,4%)	21 (38,9%)	24 (42,9%)	12 (42,9%)	89 (43,0%)
Separado	3 (4,3%)	3 (5,6%)	1 (1,8%)	1 (3,6%)	8 (3,9%)
Viúvo	2 (2,9%)	8 (14,8%)	2 (3,6%)	1 (3,6%)	13 (6,3%)
**Escolaridade**					
Ensino fundamental incompleto	25 (36,2%)	15 (27,8%)	15 (26,8%)	8 (28,6%)	63 (30,4%)
Ensino fundamental completo	10 (14,5%)	11 (20,4%)	11 (19,6%)	4 (14,3%)	36 (17,4%)
Ensino médio incompleto	6 (8,7%)	4 (7,4%)	6 (10,7%)	4 (14,3%)	20 (9,7%)
Ensino médio completo	24 (34,8%)	17 (31,5%)	20 (35,7%)	9 (32,1%)	70 (33,8%)
Ensino superior incompleto	3 (4,3%)	1 (1,9%)	2 (3,6%)	1 (3,6%)	7 (3,4%)
Ensino superior completo	1 (1,4%)	6 (11,1%)	2 (3,6%)	2 (7,1%)	11 (5,3%)
**Renda familiar mensal**					
Desempregado	0 (0,0%)	1 (1,9%)	0 (0,0%)	0 (0,0%)	1 (0,5%)
Um salário	2 (2,9%)	5 (9,3%)	0 (0,0%)	0 (0,0%)	7 (3,4%)
Dois salários	39 (56,5%)	16 (29,6%)	38 (67,9%)	15 (53,6%)	108 (52,2%)
Três salários	25 (36,2%)	15 (27,8%)	15 (26,8%)	9 (32,1%)	64 (30,9%)
Quatro salários	3 (4,3%)	11 (20,4%)	3 (5,4%)	4 (14,3%)	21 (10,1%)
Cinco salários	0 (0,0%)	4 (7,4%)	0 (0,0%)	0 (0,0%)	4 (1,9%)
Seis salários	0 (0,0%)	2 (3,7%)	0 (0,0%)	0 (0,0%)	2 (1,0%)

*UMS - Unidade Municipal de Saúde.*

Quanto às dificuldades relacionadas ao tratamento, apesar de 98 (47,3%) participantes terem afirmado que “retirar o medicamento da embalagem” não é uma tarefa difícil, 90 (43,5%) consideraram-na pouco difícil. As tarefas “ler a embalagem do medicamento”, “lembrar de tomar o medicamento” e “repor os medicamentos” foram consideradas pouco difíceis, respectivamente para 147 (71,0%), 167 (80,7%) e 105 (50,7%) participantes. Em que pese 109 (52,7%) terem afirmado que “tomar vários comprimidos ao mesmo tempo” não é uma tarefa difícil, 34 (16,4%) consideraram-na muito difícil, representando o maior percentual para essa resposta entre as dificuldades referidas (Tabela 2).

**Tabela 2:** Dificuldades relacionadas ao tratamento, referidas por pessoas em tratamento para tuberculose (N=207), Belém, Pará, Brasil, 2023

Dificuldades referidas	Muito difícil	Pouco difícil	Não é difícil
Retirar o medicamento da embalagem	19 (9,2%)	90 (43,5%)	98 (47,3%)
Ler a embalagem do medicamento	5 (2,4%)	147 (71,0%)	55 (26,6%)
Lembrar de tomar o medicamento	1 (0,5%)	167 (80,7%)	39 (18,8%)
Repor os medicamentos	18 (8,7%)	105 (50,7%)	84 (40,6%)
Tomar vários comprimidos ao mesmo tempo	34 (16,4%)	64 (30,9%)	109 (52,7%)

## DISCUSSÃO

O perfil dos participantes se constituiu, majoritariamente, por homens de 18 a 39 anos, solteiros, com baixos níveis de escolaridade e renda familiar mensal correspondente a dois salários mínimos. Reconheceram dificuldades para realizar o tratamento para TB, como retirar o medicamento da embalagem, repor os medicamentos e tomar vários comprimidos ao mesmo tempo.

O sexo predominante dialoga com a literatura, pois se expressa em dados oficiais da OMS e do Ministério da Saúde^(1,4)^, assim como em diversos estudos nacionais^(3,23)^ e internacionais^(24,25)^, ratificando maiores proporções de casos de TB nesse público. Em 2024, dos 84.308 casos novos registrados no Brasil, 57.506 (68,2%) ocorreram no sexo masculino, cujos coeficientes de incidência, quando comparados aos do sexo feminino, foram maiores em todas as faixas etárias, exceto na faixa de 10 a 14 anos^(4)^.

Focalizando a realidade local, destaca-se que, no período de 2009 a 2016, ao avaliar os casos notificados em Belém, estudo ecológico apontou 11.103 casos novos, com coeficiente de incidência igual a 97,5 casos/100 mil habitantes, idade média de 38,6 anos e desvio padrão de 17,1, evidenciando que o coeficiente de incidência foi maior no público masculino (12,4 casos/10.000 homens) do que no feminino (7,3 casos/10.000 mulheres)^(26)^.

É possível inferir que esse panorama epidemiológico guarda forte relação com a cultura relacionada à saúde do homem, pois estudos identificaram reduzida ou limitada procura desse público por serviços ofertados na APS^(27-29)^, traduzindo fator agravante para elevados índices de morbidade e mortalidade por causas diversas. Em muitas realidades, a busca de homens por esses serviços é substancialmente inferior à das mulheres, além de apresentarem baixa adesão às propostas terapêuticas e às propostas de promoção da saúde e prevenção do adoecimento^(30)^.

Assim, é notória a fragilidade do LS em grande parte do público masculino, pois pessoas que apresentam melhores níveis de LS tendem a reconhecer, com maior facilidade, a importância de ações preventivas e mantenedoras da saúde, além da adoção de práticas de autocuidado^(31)^. Entende-se que a ausência desse público nos serviços de saúde o torna mais vulnerável ao adoecimento, além de postergar diagnósticos que poderiam ser realizados precocemente e as intervenções terapêuticas correspondentes, refletindo baixa competência e habilidade do LS.

No cenário de maior adoecimento da população masculina, as limitações dos homens em frequentar os serviços de saúde podem ser explicadas por diversas razões, como os fatores históricos e socioculturais que contribuíram e ainda contribuem para produzir lógicas de pensamento antiquadas em torno da masculinidade, vinculando os homens à noção de invulnerabilidade^(29,32,33)^.

Comparando as desigualdades entre homens e mulheres no contexto da TB, a exemplo do *status* social, da posição econômica e do acesso a vários recursos, são notáveis as diferenças atribuídas aos fatores econômicos e socioculturais, influenciando a adesão ao tratamento para TB^(34)^.

Em relação à faixa etária, os resultados apontam que o público predominante apresentou entre 18 e 39 anos, aproximando-se dos dados publicados em Boletim Epidemiológico da TB, referente ao ano de 2025, que exibe os dados de incidência de 2024. Esse documento mostra que, em ambos os sexos, os maiores coeficientes de incidência foram identificados na faixa etária de 20 a 34 anos, com razão de incidência entre os sexos igual a 2,18, demonstrando que, além dos homens, os mais acometidos ocupam a faixa economicamente ativa^(4)^, como evidenciado na literatura^(35,36)^.

É válido ressaltar que pessoas mais jovens apresentam potencialidades de LS para identificar e acessar fontes de informação confiáveis, motivo pelo qual têm maior possibilidade de obter informações de qualidade e implementá-las para promover o autocuidado^(37)^. Faz-se tal ponderação considerando que, quanto maior a idade, possivelmente maiores são as fragilidades em torno do LS, pois há maior probabilidade de o envelhecimento acometer a cognição e os sentidos especiais, como audição e visão, interferindo na compreensão de informações sobre saúde e outros temas. Além disso, comumente pessoas idosas dispõem da ajuda de terceiros para realizar cuidados cotidianos, os quais recebem informações e as implementam sem que o idoso participe ativamente dos processos relativos à tomada de decisão, atribuindo-lhe, muitas vezes, papel coadjuvante no cuidado^(31)^.

Os dados referentes ao estado civil mostram que não houve grande diferença nos números de pessoas solteiras e casadas, e nos números de pessoas separadas e viúvas. Apesar disso, é possível inferir que tal variável pode refletir na mobilidade espacial da TB, ao resultar em maiores ou menores níveis de contato social com parceiros afetivo-sexuais, já que a proximidade do contato favorece a transmissão. Estudo internacional apontou que o estado civil (solteiro, viúvo ou divorciado) reflete no grau de apoio familiar durante o tratamento, fortalecendo ou reduzindo episódios de estresse psicossocial a partir das relações interpessoais^(38)^.

Pesquisa indicou associação entre TB e escolaridade em Belém, cenário no qual uma parcela dos participantes apresentou até quatro anos de estudo^(26)^. Sabe-se que pessoas menos escolarizadas expressam maior vulnerabilidade, pois apresentam maior dificuldade para acessar e compreender informações sobre saúde, resultando em implicações no LS e, consequentemente, menor habilidade para identificar riscos de transmissão da TB, além de influenciar a adesão ao tratamento^(2,39)^.

No entanto, avaliar o LS a partir do nível de escolaridade, isoladamente, não garante a precisão dos achados, visto que os anos de estudo, na educação formal, são quantificados sem considerar os conhecimentos adquiridos por meio das variadas experiências do sujeito. Como exemplo, cita-se o fato de que ele pode apresentar habilidades numéricas e de leitura e interpretação de textos, mas sentir dificuldades para implementá-las no contexto da saúde, demonstrando limitações relacionadas ao LS. Portanto, escolaridade elevada não resulta, necessariamente, em LS satisfatório^(40)^.

Outra pesquisa, realizada no mesmo município, apontou que 91% dos participantes tinham renda familiar menor que um salário mínimo^(41)^. Por sua vez, no estado da Bahia, indicou-se que a maioria das pessoas com TB (80,26%) apresentava rendimento mensal inferior a R$ 1.000,00, considerando a sua principal fonte de renda, enfatizando-se que a doença e condições de pobreza estão interligadas, fato que resulta em piores condições de saúde para essas pessoas^(42)^. Em termos de educação formal e ganhos salariais, a literatura demonstra que o nível educacional repercute nas oportunidades de emprego e, consequentemente, na renda individual e familiar. Assim, quanto mais favorável a condição socioeconômica, maiores as chances de ter LS satisfatório e boa saúde^(43)^.

Cabe salientar que a Agenda 2030 prevê, entre os 17 Objetivos de Desenvolvimento Sustentável elaborados pela Organização das Nações Unidas, a erradicação da pobreza em todas as suas formas e em todos os lugares, no intuito de criar condições necessárias para promover vida digna a todos^(7)^. Ao relacionar esses aspectos ao atual cenário multideterminado da capital paraense e aos números de casos de TB nesse território^(4)^, torna-se evidente a necessidade de propor e implementar medidas intervencionistas para além do campo biológico, abrangendo também os campos social e educacional.

Quanto às dificuldades para conduzir o tratamento, entende-se que a dificuldade de retirar o medicamento da embalagem pode ser superada a partir da iniciativa do profissional responsável pelo TDO ao auxiliar os usuários nessa tarefa que, apesar de simples, é fundamental para consolidar o tratamento, entregando os comprimidos junto ao copo de água para concretizar a ingestão. Assim, o profissional promove assistência personalizada, valorizando as limitações de cada indivíduo para atender às suas necessidades^(2)^.

A dificuldade de ler a embalagem do medicamento pode estar relacionada a fatores como menor habilidade cognitiva, restrição de leitura e complexidade das informações contidas nas embalagens^(44)^. Além disso, sabe-se que, de forma antagônica, a falta ou carência de compreensão das informações escritas pode interferir na autonomia e no empoderamento do usuário, além de colaborar para a aplicação incorreta das informações compartilhadas por profissionais de saúde, resultando em desfechos insatisfatórios e na utilização ineficaz dos serviços de saúde^(39)^.

Por fim, as dificuldades de lembrar de tomar e de repor os medicamentos podem estar relacionadas a aspectos socioeconômicos, tendo em vista o esquema terapêutico estabelecido para TB, que consiste na administração observada dos medicamentos de acordo com a rotina das UMSs, podendo ser realizada todos os dias úteis ou, pelo menos, três vezes na semana^(2,45)^. Assim, configuram dificuldades a serem enfrentadas, sobretudo, pelos usuários que exercem atividade remunerada, os quais optam, muitas vezes, por não comparecer às unidades de saúde para realizar o tratamento, em virtude do temor da perda salarial, da condição de desemprego e da consequente redução da renda familiar^(46)^.

O esquema terapêutico para TB é complexo, motivo pelo qual pode constituir uma barreira para a adesão ao tratamento quando há falta ou carência de compreensão sobre a sua importância. Nesse sentido, é evidente que o LS é fundamental para enfrentar a TB. Estudo que avaliou o LS de contatos de pessoas em tratamento para TB pulmonar identificou baixo nível de LS, concluindo que a falta ou carência de informações básicas sobre a doença configurava risco de infecção pelo *Mycobacterium tuberculosis* e possível adoecimento^(13)^. Outro estudo demonstrou que pessoas com baixos níveis de LS estão mais suscetíveis à não adesão medicamentosa, pois esses níveis guardam relação com o fato de não se compreender informações sobre saúde^(47)^.

Ao analisar as dificuldades relatadas pelos participantes deste estudo, percebeu-se a necessidade de implementar estratégias que minimizem as possíveis repercussões causadas por tais dificuldades. Nesse sentido, o enfermeiro tem papel crucial para ajudar os usuários a superá-las, uma vez que é reconhecido pela capacidade de compreender holisticamente o ser humano, atuar com foco na integralidade da assistência e acolher fragilidades para transformá-las em potencialidades^(9)^.

Diversos estudos relataram a participação ou as contribuições da enfermagem no fortalecimento das ações de controle da TB, entre as quais se destaca a assistência às pessoas com a doença^(48-51)^. Ampla revisão de escopo, que incluiu 40 estudos nacionais e internacionais, identificou as ações de enfermagem que promovem a adesão ao tratamento para TB, organizando duas categorias temáticas: uma sobre os cuidados direcionados às necessidades específicas dessas pessoas; e outra sobre o enfrentamento dos determinantes sociais da saúde pelos profissionais de enfermagem^(52)^.

Caracterizadas por preparar suas equipes multiprofissionais e monitorá-las para promover o LS, as Organizações Letradas em Saúde estimulam a implementação de estratégias que, ao envolver usuários, profissionais e instituições, direcionam cuidados centrados no indivíduo, aumentando sua efetividade. Sabe-se que o atendimento às demandas de forma individualizada é essencial para conduzir o tratamento, pois estabelece vínculos e confiança, gerando resultados de saúde positivos^(9,39)^. Assim, para promover o LS, é necessário que o enfermeiro se aproprie de estratégias adequadas, como a comunicação clara e objetiva, além da possibilidade de usar tecnologias de fácil aplicação^(39)^.

Isso demonstra que o fato de conhecer dificuldades relacionadas ao tratamento para TB é essencial, pois possibilita refletir criticamente e propor ações coordenadas entre gestores, profissionais de saúde e usuários, sendo direcionadas para atender às necessidades individuais e coletivas, contribuindo para evitar a interrupção terapêutica e fortalecer o controle da doença^(47)^.

### Limitações do estudo

As limitações estão relacionadas ao fato de o estudo ter se desenvolvido em unidades de saúde da APS localizadas em apenas três distritos no município de Belém, configurando realidade específica da atenção à saúde. As características epidemiológicas, geográficas, socioeconômicas e territoriais podem ter influenciado substancialmente os dados, por serem fatores que se relacionam com a produção de saberes e práticas, repercutindo, assim, no LS para fortalecer ou reduzir as dificuldades em torno do tratamento para TB.

Para ampliar as evidências sobre o tema e melhor compreendê-lo, é fundamental que novos estudos sejam realizados, visando abranger maior número de unidades e outros distritos nesse município, além de maior número de municípios na região Norte e em outras regiões do território nacional. Essa consideração visa incorporar os outros níveis de atenção à saúde, tendo em vista que pessoas com TB são atendidas e tratadas na APS, na atenção secundária/especializada e na atenção terciária/hospitalar, de acordo com as características clínicas que apresentam e com as necessidades de suporte terapêutico que advêm dessas características.

### Contribuições para as áreas de enfermagem, saúde ou políticas públicas

Diante da sua relevância, entende-se que este estudo contribui para as áreas de enfermagem e saúde coletiva, na medida em que revela o perfil de pessoas com TB e dificuldades em torno do tratamento da doença. Assim, apontou a necessidade de atualizar, reestruturar ou reforçar saberes e práticas dos usuários por meio de ações e estratégias educacionais no cotidiano da atenção à saúde, visando fortalecer o LS como elemento fundamental para enfrentar e superar essas dificuldades.

Isso demonstra que conteúdos inerentes ao LS devem transcorrer os processos formativos dos estudantes na educação superior, a exemplo dos estudantes de enfermagem, e os processos formativos dos profissionais na educação continuada e na educação permanente, a exemplo das equipes de enfermagem, principalmente dos enfermeiros. Eles devem liderar essas equipes com responsabilidade ética, científica, técnica e social, relacionando-se fortemente com os demais integrantes das equipes interdisciplinares e multiprofissionais, como norteiam a universalidade, a integralidade e a equidade, princípios doutrinários do SUS.

Os resultados e as discussões que deles sucederam podem mobilizar reflexões entre estudantes, profissionais e gestores, autoridades públicas, usuários dos serviços de saúde e outros seguimentos da sociedade civil organizada. Tal mobilização constitui oportunidades nas quais políticas públicas de saúde e educação podem ser propostas ou repensadas, sobretudo nos âmbitos local ou regional, para qualificar a assistência aos grupos humanos, a gestão desses serviços, o ensino, a pesquisa e a participação política em esferas de governança e em esferas de tomada de decisão que afetam esses grupos.

## CONCLUSÕES

Identificou-se que o perfil sociodemográfico foi especialmente constituído por pessoas do sexo masculino, na faixa etária de 18 a 39 anos, solteiras, com baixa escolaridade e baixa renda familiar mensal. No tratamento para TB, foram evidenciadas dificuldades como retirar o medicamento da embalagem, repor os medicamentos e tomar vários comprimidos ao mesmo tempo, apontando que investimentos em recursos diversos, como recursos estruturais, humanos e materiais, devem ser realizados por profissionais e gestores dos serviços de saúde e por autoridades públicas, de acordo com sua governabilidade, visando qualificar a APS.

Esses investimentos precisam fortalecer o LS dos usuários, para que os saberes e as práticas que desenvolvem no cotidiano potencializem as chances de obter a cura e controlar a doença tanto no município de Belém quanto, de forma abrangente, no cenário amazônico e no território nacional. Faz-se tal ponderação considerando que, apesar da evolução do conhecimento técnico-científico e de ser alvo de políticas e programas de saúde ao longo dos anos, a TB ainda assola indivíduos e grupos, limitando sua qualidade de vida e as possibilidades para melhorá-la.

Por essa razão, longo caminho ainda deve ser galgado para superar a TB como relevante problema de saúde pública, contexto no qual o LS expressa possibilidades robustas para que essas pessoas reflitam sobre as suas condições de vida e saúde e a necessidade de transformar, em algum grau, as maneiras como pensam e agem, como obtém informações sobre saúde, como as valorizam e avaliam sua pertinência, como as aplicam e compartilham, e as explicações que fundamentam e justificam tais maneiras e as escolhas em seu entorno.

Investimentos que viabilizem conhecer o perfil sociodemográfico de pessoas com TB, em diferentes realidades sociais e territoriais, são fundamentais para caracterizar as necessidades individuais e coletivas que advém desse perfil. Isso possibilita melhor direcionar o controle da doença nos municípios e estados da federação, sobretudo naqueles cujas características geográficas implicam grandes desafios, como é o caso particular dos municípios e estados da região amazônica.

Parte das ações e estratégias de controle deve se concentrar em fortalecer o LS dos usuários, mediante orientações profissionais qualificadas e o acesso democrático a produtos tecnológicos que guiem processos de educação em saúde na APS. Isso tem por intuito capacitar pessoas com TB para que desenvolvam seu autocuidado de forma racional, sustentável e dialógica, isto é, baseado em atitudes critico-reflexivas que resultem da interação profícua entre elas e os profissionais.

Essa interação deve não apenas contribuir para o compartilhamento de saberes, práticas e experiências, mas também para fortalecer tanto os diálogos entre conhecimento científico e senso comum quanto os vínculos que geram confiança e comunicação, pois são aspectos que entremeiam o LS e influenciam fenômenos relacionados à saúde. Portanto, com ações e estratégias que valorizem o LS e a dimensão coletiva do adoecimento, entende-se que os sujeitos possam melhor enfrentar a TB como doença socialmente determinada, ressaltando a importância de estudos como este.

## Data Availability

Os dados de pesquisa só estão disponíveis mediante solicitação.
